# Use of outpatient care in VA and Medicare among disability-eligible and age-eligible veteran patients

**DOI:** 10.1186/1472-6963-12-51

**Published:** 2012-03-05

**Authors:** Chuan-Fen Liu, Chris L Bryson, James F Burgess, Nancy Sharp, Mark Perkins, Matthew L Maciejewski

**Affiliations:** 1 Northwest Center for Outcomes Research in Older Adults, VA Puget Sound Health Care System, 1100 Olive Way, Suite 1400, Seattle, WA 98101, USA; 2 Department of Health Services, University of Washington, Seattle, USA; 3 Division of General Internal Medicine, Department of Medicine, University of Washington, Seattle, USA; 4 Center for Organization, Leadership & Management Research, VA Boston Healthcare System, Boston, USA; 5 Department of Health Policy and Management, School of Public Health, Boston University, Boston, USA; 6 Center for Health Services Research in Primary Care, Durham VA Medical Center, Durham, USA; 7 Division of General Internal Medicine, Department of Medicine, Duke University Medical Center, Durham, USA

**Keywords:** Outpatient, Utilization, Primary care, Veterans, Medicare

## Abstract

**Background:**

More than half of veterans who use Veterans Health Administration (VA) care are also eligible for Medicare via disability or age, but no prior studies have examined variation in use of outpatient services by Medicare-eligible veterans across health system, type of care or time.

**Objectives:**

To examine differences in use of VA and Medicare outpatient services by disability-eligible or age-eligible veterans among veterans who used VA primary care services and were also eligible for Medicare.

**Methods:**

A retrospective cohort study of 4,704 disability- and 10,816 age-eligible veterans who used VA primary care services in fiscal year (FY) 2000. We tracked their outpatient utilization from FY2001 to FY2004 using VA administrative and Medicare claims data. We examined utilization differences for primary care, specialty care, and mental health outpatient visits using generalized estimating equations.

**Results:**

Among Medicare-eligible veterans who used VA primary care, disability-eligible veterans had more VA primary care visits (*p *< 0.001) and more VA specialty care visits (*p *< 0.001) than age-eligible veterans. They were more likely to have mental health visits in VA (*p *< 0.01) and Medicare-reimbursed visits (*p *< 0.01). Disability-eligible veterans also had more total (VA+Medicare) visits for primary care (*p *< 0.01) and specialty care (*p *< 0.01), controlling for patient characteristics.

**Conclusions:**

Greater use of primary care and specialty care visits by disability-eligible veterans is most likely related to greater health needs not captured by the patient characteristics we employed and eligibility for VA care at no cost. Outpatient care patterns of disability-eligible veterans may foreshadow care patterns of veterans returning from Afghanistan and Iraq wars, who are entering the system in growing numbers. This study provides an important baseline for future research assessing utilizations among returning veterans who use both VA and Medicare systems. Establishing effective care coordination protocols between VA and Medicare providers can help ensure efficient use of taxpayer resources and high quality care for disabled veterans.

## Background

The Medicare program and the health care system of Department of Veterans Affairs (VA) are the two major public health care programs funded by the federal government of the United States (US).

The Medicare program, the largest public health program in the US, provides health care for 47 million elderly and disabled Americans, with an annual budget of $510 billion in 2010. The majority of Medicare beneficiaries are in the fee-for-service Medicare plans. Individuals under 65 can become disability-eligible for Medicare if they have sufficient work history for their age and receive Social Security Disability Insurance (SSDI) for 24 months, develop end-stage renal disease (ESRD) with a 3-month waiting period, or have amyotrophic lateral sclerosis/Lou Gehrig's disease and receive SSDI (no waiting period). About 14% of Medicare enrollees were disability-eligible beneficiaries in 2004 [1] and that proportion was expected to grow to 17% by 2010 [2].

The VA health care system, the largest integrated health care system in the US, provides comprehensive medical care to veterans with a budget of $48 billion in 2010. Eligibility for VA health care benefits is determined by a system of eight Priority Groups based on the factors including the level of service-connected injuries and conditions and incomes. The VA system had 153 hospitals and more than 900 outpatient clinics, served 5.5 million users, and provided 60 million outpatient visits in 2008. More than half of veterans who use VA are also eligible for Medicare [3] due to disability or age. In 2002, 16% of the 3.4 million of Medicare-eligible veterans in VA were disability-eligible Medicare beneficiaries under 65 years old [4]. The VA health care system has been serving as a safety net to disabled and low income veterans for some time [5,6], and it is likely that many veterans returning from Afghanistan and Iraq will rely upon VA as a usual source of care because they are eligible for VA care at no expense for five years.

Compared to the general non-elderly U.S. population, non-elderly people with disabilities are less likely to be employed, more likely to have poor or fair self-reported health status, and have lower incomes [1,7,8]. People with disabilities have been shown to be less likely to receive primary preventive care, have difficulty paying for inpatient and outpatient health services and medications, and have more preventable emergency room visits and hospitalizations than the general population [7-10].

While some prior research has examined use of Medicare and Medicaid services by disability-eligible beneficiaries [7,11-14], much less research has been conducted on disability-eligible beneficiaries than age-eligible beneficiaries [15,16]. In 2000, based on the census data [17] and VA administrative data, 9.6% of 16.7 million of veterans under 65 years old used the VA system, compared to 16.5% of 9.7 million veterans over 65 years old used the VA system.

Compared to age-eligible Medicare beneficiaries, disability-eligible beneficiaries are more likely to be in poorer health and to have cognitive or mental health impairments [1,13,18] which can be the proximal reason for the disability-eligibility. Research also shows that disability-eligible beneficiaries incur higher average per capita Medicare expenditures than age-eligible beneficiaries [18], and have disproportionally higher total Medicare expenditures [15]. A recent study shows that disability-eligible beneficiaries experience more problems with health care cost and access to care than age-eligible beneficiaries [19]. Further, disability-eligible beneficiaries report lower levels of satisfaction with care and more barriers to health care than age-eligible beneficiaries [7,16,20-22].

Research on Medicare-eligible veterans has focused almost exclusively on age-eligible veterans. Age-eligible veterans obtain a significant proportion of their inpatient care outside the VA [23-29]. A study using a VA national sample of elderly Medicare-eligible veterans found that 36% used outpatient services funded by Medicare and 46% used both Medicare and VA outpatient services in 1999 [28]. A study examining 1997-1999 outpatient care patterns for male, elderly Medicare-eligible veterans from three states in New England found that these veterans tended to use VA for specialty and mental health care, but relied on Medicare for primary care [27]. A recent study that pooled disability- and age-eligible VA primary care users found that more than 30% used primary care in Medicare and more than 60% used specialty care in Medicare [30]. However, this study did not compare differences in outpatient use between disability- and age-eligible veterans.

Petersen and colleagues [31] found that VA reliance, measured as the proportion of 2004 medical expenditures incurred in VA to total (VA+Medicare) expenditures, was higher in the age under 65 group than the over 65 group (0.8 versus 0.53). VA reliance based on utilization, as measured in this study, may provide a different picture of outpatient utilization patterns but since costing methodologies differ considerably between VA and Medicare may provide a more stable dependent variable for analysis [32]. Furthermore, most studies that differentiate disability-eligible and age-eligible beneficiaries on the basis of age misclassify veterans who are age over 65 as age-eligible veterans when their original reason for Medicare eligibility was disability. In this current work we identify disability-eligible veterans based on their original reason for Medicare-eligibility. Finally, it is important to examine outpatient utilization patterns, because chronic disease management occurs in outpatient care settings. The VA system serves a veteran population with a much greater prevalence of chronic illness than general populations [33]. Medicare provides veterans with increased choice, access, and flexibility in their health care [27,34,35] and may provide access to services unavailable in their local VA system [27,34,36,37]. However, continuity and coordination of care may suffer if Medicare-eligible veterans seek outpatient care in both systems, especially for individuals with chronic conditions requiring on-going and effective management.

This paper examined differences in outpatient utilization in VA and Medicare between disability-eligible and age-eligible veterans who used VA primary care services. Based on prior work in non-veteran samples [7-10,15], we expected disability-eligible veterans to have greater outpatient utilization than age-eligible veterans. We also expected both groups to have more mental health visits in VA than Medicare [38]. This study didn't include Medicare-eligible veterans who solely relied on Medicare.

The results of this analysis can inform VA and Medicare policy by identifying the outpatient services in each public insurance system that may be in great demand by current and future disability-eligible veterans and by identifying Medicare-eligible veterans who will require improved coordination of care to minimize duplication and contain federal health expenditures [38]. As soldiers returning from Afghanistan and Iraq with disabilities transition into the VA system, this study provides an important baseline estimate with which to compare outcomes of disability-eligible veterans who served in these two wars and insights into challenges and potential resource needs for caring for current and future disability-eligible veterans.

## Methods

This was a retrospective cohort study conducted using administrative data covering Fiscal Year (FY) 2000-2005 (10/1/1999-9/30/2005). We identified the patient cohort, who were VA primary care users and also eligible for Medicare in the baseline year of FY2000 and tracked their outpatient utilization obtained from both VA and Medicare sources for the four subsequent years (FY2001-FY2004).

### Sample and data

The sample was drawn from a prior study [39] of 66,366 elderly and non-elderly veterans from 72 VA medical centers and 108 community clinics located in all states in the US except Alaska. The sampling frame for this study consisted of veterans who had primary care visits in a VA medical center or community clinic in FY2000. Medicare-eligible veterans, who solely relied on Medicare, were not included in the study.

Additional inclusion and exclusion criteria were applied for this analysis. First, we excluded veterans who died before the end of FY2001, the first year of the follow-up period for the study (n = 4,033). We identified deaths using dates of death from VA and Medicare data. Second, we excluded those who did not enrolled in Medicare or were Medicare-enrolled but without both Medicare Parts A (inpatient coverage) and B (outpatient coverage) during the entire study period (n = 33,360) as indicated by the Medicare entitlement/buying indicator of the Medicare Denominator files. The requirement of both Part A and Part B data was because this analysis focused on outpatient services. Third, we excluded veterans who developed end stage renal disease (ESRD) and became eligible for the Medicare ESRD program prior to or during the study period, 2000-2004 (n = 422), because their benefits under the Medicare ESRD program and their health needs were likely to be significantly different from non-ESRD patients. ESRD status was determined by the ESRD code in the Medicare status indicator, current reason for entitlement, or original reason for entitlement variable in the Medicare denominator files. Fourth, we excluded all Medicare managed care enrollees using the Health Maintenance Organization (HMO) enrollment indicator in the Medicare Denominator files (n = 5,506), because Medicare databases do not collect service utilization data on these patients. Finally, we excluded Medicare-eligible veterans who did not use primary care in VA in FY2000 (n = 7,525) as defined below. After applying these exclusion criteria, the final sample included 15,520 Medicare-enrolled veterans located in 49 of 50 US states who used VA primary care in 2000 with 6,556 hospital-based primary care users and 8,964 community-based primary care users. The criteria for the selection of community clinics and VA hospitals in the prior study [39] led to a sampling process for this study sample that was not entirely nationally representative. Our study sample was similar to a national sample of Medicare-eligible veterans using VA care [4], but was slightly younger, more likely to be white, lived farther away from a VA hospital or community clinic, and had somewhat fewer individuals covered by Medicaid.

We defined disability- versus age-eligible based on the original reason for Medicare eligibility. This definition of disability included veterans, who were 65 years or older in FY2000, who became Medicare-eligible because of disability before 65 years old.

Data sources included 1999-2004 Medicare claims data, FY2000-FY2004 VA administrative data and 2000 Census data. We identified primary care, specialty care and mental health care utilization outcomes from FY2001-FY2004 VA Outpatient Care Files and Medicare Carrier Standard Analytical Files. Medicare Denominator files were used to identify Medicare and Medicaid eligibility and managed care enrollment. We obtained patient characteristics at baseline (2000) from Medicare Denominator files and VA Outpatient Care files. Finally, we used 2000 US Census data for the ZIP level characteristics where the patient lived.

### Measures

#### Number of visits by type of outpatient care

The outcomes of interest in this study were the number of visits for three types of outpatient care: primary care, specialty care, and mental health in VA and Medicare in each year from FY2001-FY2004. To compare outpatient use between VA and Medicare, we developed a classification algorithm for different types of outpatient care by using provider specialty and CPT procedure codes that are available in both systems. This approach allows us to construct comparable visits by type of outpatient care from two data systems, which have different data generating processes. The classification algorithm is described in detail in the paper by Burgess and colleagues [32]. The outpatient visits included face-to-face encounters with selected providers, including physicians, nurse practitioners, physician assistants, and non-physician mental health providers (psychologists and social workers). Provider specialties were classified into three categories: primary care, specialty care, and mental health care. Then, we classified CPT procedure codes for each visit into general categories: anesthesia, evaluation/management (E/M), medicine, psychiatry, and surgery. We further classified E/M CPT codes into primary care and specialty care. Finally, based on the combination of provider specialty and procedure codes, we classified each face-to-face encounter into one of the three types of outpatient care: primary care, specialty care, and mental health care. We classified patients into four mutually exclusive groups for each type of outpatient care in each year: VA use only, Medicare use only, dual use of VA and Medicare, and no use.

#### Independent variable

The independent variable of interest is whether a veteran was disability- or age-eligible for Medicare based on the original reason for Medicare eligibility.

#### Control variables

We controlled for other patient level covariates at the baseline year (FY2000) based on the theoretical framework described by the Behavioral Health model for vulnerable populations [40] that health care utilization is influenced by predisposing, enabling, and need factors. Predisposing factors included age, sex, race, and marital status. We obtained race information from both VA and Medicare data since there is incomplete race data in the VA administrative database.

Enabling factors included both the patient level and community level measures at the baseline year (FY2000). Patient characteristics included Medicaid coverage, veteran's VA copayment status, type of VA clinic where a patient received primary care (community versus hospital primary care clinic), and the distance from patient's residence to the closest VA facility (hospital or community clinic). We classified VA copayment status for each veteran into three groups: 1) receiving free care in VA due to a military service-related disability at 50% or higher, 2) receiving free care in VA due to low income, and 3) not eligible for free care in VA and required to pay copayments ($50 for a specialty care visit, $15 for a primary care visit, and $8 for a 30-day supply of medication). Three community level measures were used as potential measures of resource availability of the community where the patient resides, including the median income at the ZIP code level, the proportion of high school graduates at the ZIP code level, and the population density at the county level.

The need factors were the military service related disability and the Diagnostic Cost Group (DCG) risk adjustment measure that accounts for multiple comorbid conditions. The DCG system is a widely used diagnosis-based case-mix adjustment system based on both inpatient and outpatient diagnoses obtained from administrative files or claims databases [41]. The DCG model assigns ICD-9-CM diagnosis codes to disease groups that are clinically related and show similar levels of resource use. Multiple conditions are allowed in the DCG model to account for comorbidities. The DCG predicts VA costs, utilization, and mortality better compared to other risk adjusters [42-45]. The DCG model has been used to adjust for Medicare capitation payments [46] and is currently used for Medicare inpatient performance measures, such as mortality or hospital readmissions. The DCG model generates a standardized summary score. A DCG score greater than 1 indicates that the case-mix is greater than the average of the reference population, while a DCG score less than 1 indicates the case-mix is lower than the average. Our study constructed the DCG using the Medicare population as the reference population.

### Analysis

Descriptive and bivariate statistics, including t-tests and chi-squared tests, assessed differences in patient characteristics between disability-eligible and age-eligible veterans in the cohort identification year (FY2000). The unit of analysis for utilization comparisons was the person year. Due to the skewness of utilization data, we used Wilcoxon nonparametric rank-sum tests to compare unadjusted differences in utilization. For multivariate analysis, we estimated the annual number of primary care and specialty care visits in FY2001-2004 using generalized estimating equations (GEEs) with a negative binomial distribution and a log link function.

We used longitudinal two-part models for mental health visits because a significant proportion of veterans did not use mental health care. The first part of the model uses GEE logistic regressions to estimate the odds of having any mental health visit. The second part uses the GEE negative binomial regression, described above, to estimate the number of mental health visits among veterans who had one or more mental health visits. We employed the independent-correlation and the robust option.

The multivariate analysis adjusted for patient characteristics, zip code level income and education, county-level population density and year fixed effects. Incidence rate ratios (IRRs) and differences in the predicted number of outpatient visits were generated to examine whether VA, Medicare and total (VA+Medicare) outpatient visits differed between disability-eligible and age-eligible veterans. Significance was lowered from 0.05 to 0.01 to adjust for multiple comparisons.

All analyses were weighted for the sampling strategy used by the original community clinic evaluation study [39]. Analyses were conducted using Stata 11 [47]. Human subjects approvals for these analyses were obtained from Institutional Review Boards at the Durham VA Medical Center, VA Boston Healthcare System, and VA Puget Sound Health Care System.

## Results

### Patient characteristics

Compared to age-eligible veterans, disability-eligible veterans were younger (61 years versus 74 years, *p *= 0.001), more likely to be enrolled in Medicaid (8% versus 4%, *p *< 0.0001), more likely to receive free care in VA (overall: 92% versus 79%, p < 0.0001) due to disability (44% versus 33%, *p *< 0.0001) or low income (48% versus 44%, *p *= 0.0007), and had a higher level of service-related disability (28 versus 13, p < 0.001) (Table 1). Over 40% of disability-eligible veterans were 65 years or older. Compared to age-eligible veterans, disability-eligible veterans were less likely to be married (59% versus 71%, *p *< 0.0001), white (87% versus 91%, *p *< 0.0001), or receive primary care in community clinics (73% versus 78%, *p *= 0.001). For community characteristics, compared to age-eligible veterans, disability-eligible veterans were more likely to live in areas with lower per capita incomes (*p *= 0.001) or a lower percentage of high school graduates (*p *= 0.001).

**Table 1:** Baseline Characteristics among VA and Medicare Dual Users in FY2000: Disability-versus Age-Eligible Veteran Patients^1^

	Disability (n = 4,704)	Age (n = 10,816)	p-value
**Patient Characteristics**			

Age, years	61.4	73.8	0.001

Age > 65 (%)	43.2%	100%	< 0.0001

Sex: female (%)	3.3%	3.4%	0.0297

Married (%)	59.3%	71.1%	< 0.0001

White (%)	87.1%	91.2%	< 0.0001

Medicaid eligibility (%)	7.9%	3.8%	< 0.0001

Receiving free VA care (%)	92.4%	76.8%	< 0.0001

Due to disability (%)	44.2%	33.1%	< 0.0001

Due to low income (%)	48.2%	43.7%	0.0007

Service-related disability percentage	28.1	13.4	0.001

Diagnostic cost group (DCG) Score^2^	0.98	0.95	0.104

Receiving primary care at VA community primary care clinic (%) (ref: hospital-based primary care users)	73.2%	77.6%	0.001

Distance from patient's residence to the closest VA facility^3^, miles	21.4	20.9	0.568

**Community Characteristics**			

Per capita income in zip code	18,703	19,843	0.001

Population per sq mile in county	666	917	0.040

Percentage high school graduate in zip code	78.2	80.2	0.001

^1^Disability- or age-eligible for Medicare was defined based on the original reason for entitlement. All comparisons were adjusted for sampling weights.

^2^Measured at FY2000 including diagnoses from both VA and Medicare.

^3^A VA hospital or a community clinic.

### Veterans' use of primary care in Medicare and VA

**Figure 1:**
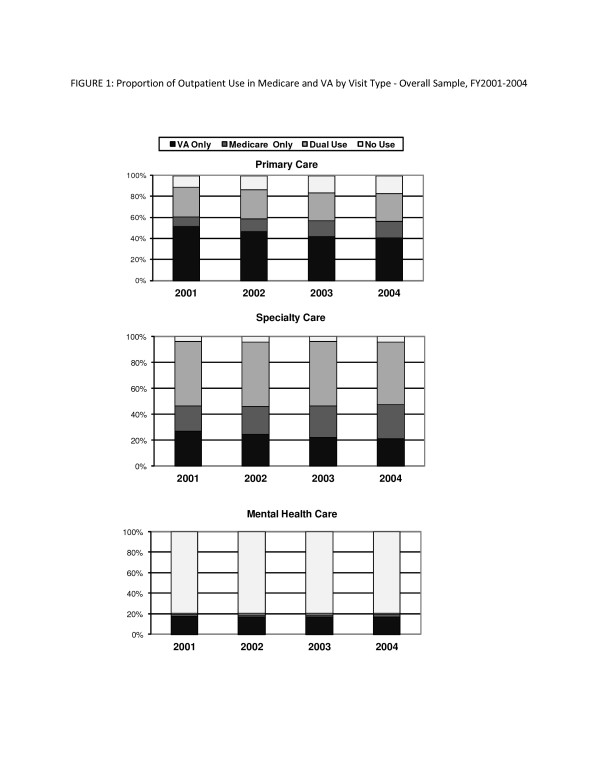
**Proportion of Outpatient Use in Medicare and VA by Type of Outpatient Care- Overall Sample, FY2001-2004**.

For the overall study sample, the largest proportion of Medicare-eligible veteran patients used primary care only in VA, but this proportion declined over time, from 51.7% in FY2001 to 40.9% in FY2004 (Figure 1, top panel). The proportions of veterans obtaining primary care only in Medicare increased over time, from 8.8% in FY2001 to 15.4% in FY2004, while the proportion of veterans using primary care in both VA and Medicare were stable over time (26-28%).

**Figure 2:**
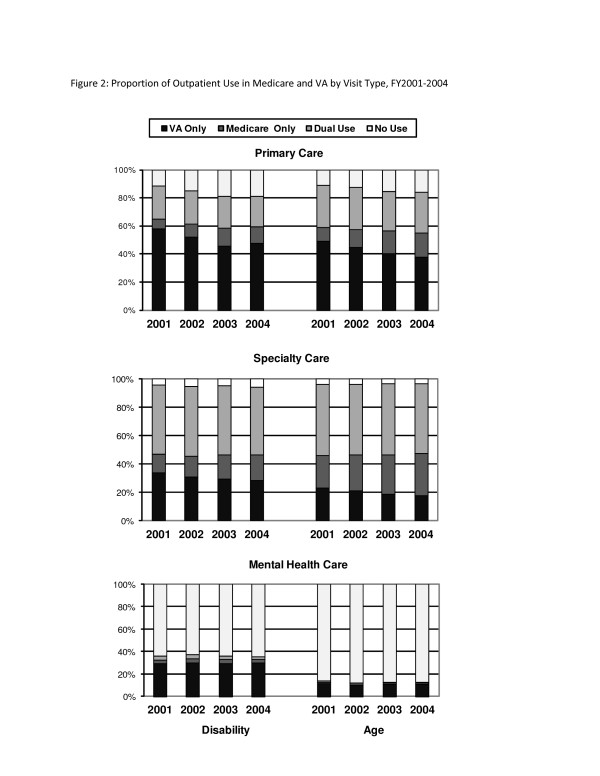
**Proportion of Outpatient Use in Medicare and VA by Type of Outpatient Care, FY2001-2004**.

For both disability-eligible veterans and age-eligible veterans, the largest proportion of these veterans used primary care only in VA, but this proportion declined over time (Figure 2, top panel). Disability-eligible veterans were more likely than age-eligible veterans to obtain primary care only at the VA (disability-eligible: 46-58%, age-eligible: 38-49%; *p *< 0.001 for all years). The proportion of veterans using primary care in both VA and Medicare remained stable over time (disability-eligible: 22-24%, age-eligible: 28-30%), and disability-eligible veterans were less likely to use both VA and Medicare primary care in all years. The proportions of veterans obtaining primary care only in Medicare increased over time, but in all years disability-eligible veterans were less likely than age-eligible veterans to receive primary care only in Medicare (disability-eligible: 7-13%, age-eligible: 10-17%; *p *< 0.001 for all years).

**Figure 3:**
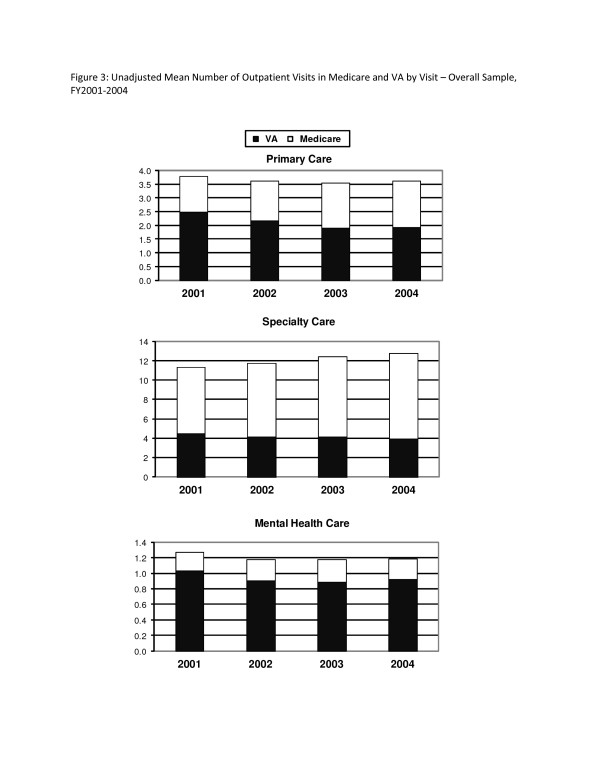
**Unadjusted Mean Number of Visits in Medicare and VA by Type of Outpatient Care - Overall Sample, FY2001-2004**.

For the overall sample, the total number of primary care visits was stable over time (3.8-3.5 visits) (Figure 3, top panel). The number of VA primary care visits decreased over time (2.5 visits in 2001 and 1.9 visits in FY2003-2004), while use of primary care financed by Medicare increased over time (1.3 visits in FY2001 and 1.7 visits in FY2003-2004).

**Figure 4:**
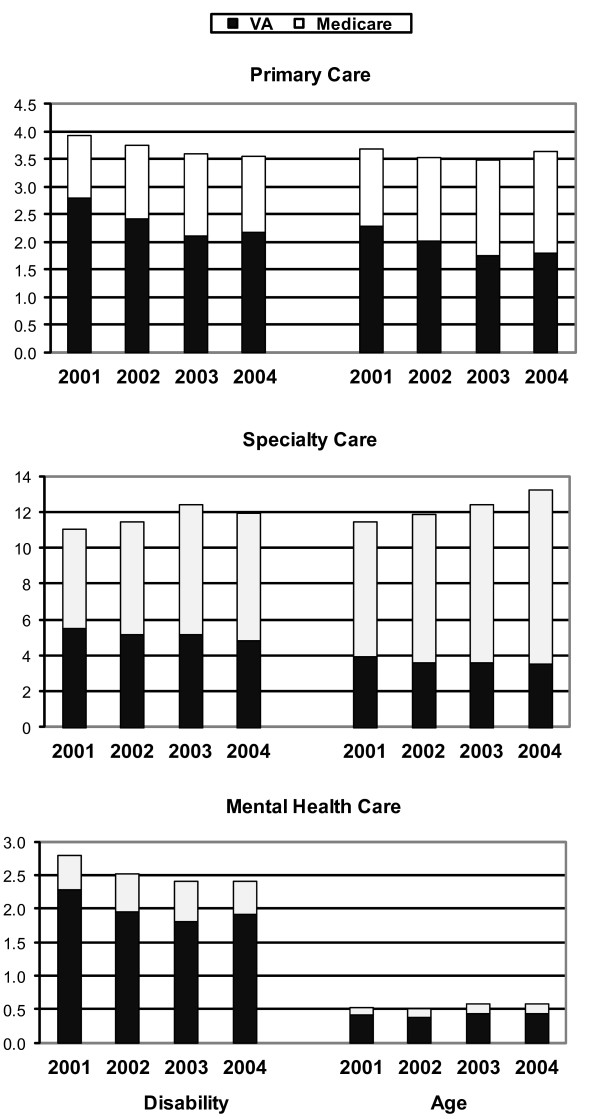
**Unadjusted Mean Number of Visits in Medicare and VA by Type of Outpatient Care, FY2001-2004**.

Disability-eligible veterans had more VA primary care visits than age-eligible veterans (2.1- 2.8 vs. 1.8-2.3; *p *< 0.001 for all years), but fewer primary care visits financed by Medicare (1.1-1.5 vs. 1.4-1.8; *p *< 0.001 for all years). Use of VA primary care decreased in both groups over time, while use of Medicare primary care services increased over time (Figure 4, top panel).

### Veterans' use of specialty care in Medicare and VA

For the overall study sample, half of Medicare-eligible veteran patients used specialty care in both VA and Medicare and this proportion remained stable over time (Figure 1, middle panel). The proportions of veterans obtaining specialty care only in VA decreased over time from 26% in FY2001 to 21% in FY2004, while the proportion of veterans obtaining specialty care in only Medicare increased over time from 20% in FY2001 to 26% in FY2004.

The patterns of specialty care use from the overall sample were also observed in both disability- and age-eligible veterans, respectively (Figure 2; middle panel). Disability-eligible veterans were more likely than age-eligible veterans to obtain specialty care only in VA (disability-eligible: 28-34%, age-eligible: 17-23%; *p *< 0.001 for all years), but the proportions of both disability- and age-eligible veterans obtaining specialty care only in VA decreased over time. About half of veterans used specialty care in both VA and Medicare and this proportion remained stable over time for both disability- and age-eligible veterans. The proportion of veterans obtaining specialty care only in Medicare increased over time, but disability-eligible veterans were less likely to obtain specialty care only in Medicare in all years (13-18% vs. 23-30%; *p *< 0.001 for all years).

For the overall sample, the total number of specialty care visits increased over time, from 11.3 visits in FY2001 to 12.8 visits in FY2004 (Figure 3, middle panel). The number of VA specialty care visits decreased over time from 4.4 visits in FY2001 to 3.9 visits in FY2004, while use of specialty care financed by Medicare increased over time from 6.9 visits in FY2001 to 8.8 visits in FY2004).

Disability-eligible veterans had more VA specialty care visits than age-eligible veterans (4.8- 5.5 vs. 3.5-3.9, *p *< 0.001) in FY2001-4, fewer Medicare specialty care visits (5.5-7.1 vs. 7.5-9.7, *p *< 0.001) and fewer total specialty care visits (11.0-12.4 vs. 11.4-13.2, *p *< 0.001) (Figure 4, middle panel). Use of VA specialty care decreased in both groups over time, while use of Medicare specialty care services increased over time.

### Veterans' use of mental health care in Medicare and VA

The utilization patterns for mental health care use were stable over time for the overall study sample (Figure 1, bottom panel). The majority of Medicare-eligible veteran patients did not use any outpatient mental health care (80% across years). For the overall study sample, 16-17% of used mental health care only in VA, 2% used care only in Medicare, and 1-2% used both VA and Medicare.

The patterns of mental care use from the overall sample were also observed in disability- and age-eligible veterans, respectively (Figure 2; bottom panel). The majority of disability-eligible veterans (64-65%) and age-eligible veterans (87-88%) did not use any outpatient mental health services in VA or Medicare. Disability-eligible veterans were three times more likely to use outpatient mental health services than age-eligible veterans and these proportions remained stable over time. Veterans who used mental health services were much more likely to obtain mental health care only in VA, and disability-eligible veterans obtained mental health services in VA at much greater rates than age-eligible veterans (29-30% vs. 10-11%). A very small proportion of disability- and age-eligible veterans used outpatient mental health care only in Medicare (disability-eligible: 2.9-3.3%; age-eligible: 1-2%) or in both VA and Medicare (disability-eligible: 2.6-3.4%; age-eligible: 0.4-1%).

Finally, the total number of specialty care visits for the overall sample remained stable over time (1.2-1.3 visits) with the majority of visits occurred in VA (0.9-1.0 visits) (Figure 3, bottom panel). Disability-eligible veterans had more total mental health care visits than age-eligible veterans (2.8 vs. 0.5, *p *< 0.001) in FY2001, with the majority of visits occurring in VA for both groups (2.3 vs. 0.4, *p *< 0.001) (Figure 4, bottom panel). Disability-eligible veterans had more Medicare mental health visits than age-eligible veterans in all years.

### Adjusted number of visits

After adjusting for patient characteristics, zip code-level characteristics and year fixed effects (Table 2), disability-eligible veterans had 11% (0.8 visits) more VA primary care visits (aIRR = 1.11, *p *< 0.001) than age-eligible veterans and 7% (0.6 visits) more total (VA+Medicare) visits (aIRR = 1.07, *p *< 0.01), but similar numbers of primary care visits in Medicare (*p *= 0.84). Disability-eligible veterans had 19% (2.5 visits) more specialty care visits in VA (aIRR = 1.19, *p *< 0.0001) and 7% (0.6 visits) more total specialty care visits (aIRR = 1.07, *p *< 0.01) than age-eligible veterans, but similar numbers of specialty care visits in Medicare (*p *= 0.82).

**Table 2:** Adjusted Annual Primary Care and Specialty Care Visits between Disability- and Age-Eligible Veteran Patients

Disability eligible (reference = age eligible)		
	
Utilization	**Incidence rate ratio**^**1**^ (95% CI)	Difference in predicted **visits per year**^**2**^
Primary care		

VA	1.11 (1.05-1.18)**	0.81

Medicare	1.01 (0.91-1.12)	-0.30

Total	1.07 (1.02-1.13)*	0.55

Specialty care		

VA	1.19 (1.11-1.29)**	2.47

Medicare	0.99 (0.92-1.07)	-1.92

Total	1.07 (1.02-1.12)*	0.61

*p < 0.01; **p < 0.001

^1^Adjusted for patient characteristics, zip code level characteristics, year fixed effects, and sampling weights.

^2^The predicted number of visits was calculated using the regression coefficients and patient characteristics of a typical veteran in FY2003. A typical veteran patient was defined using the mean values of all continuous patient variables and the modal values of all dichotomous patient variables.

Disability-eligible veterans were more likely than age-eligible veterans to have any VA mental health visits (Odds Ratio (OR) = 1.74, *p *< 0.001), any Medicare mental health visits (OR = 1.46, *p *< 0.01), and any mental health care visits in either system (OR = 1.73, *p *< 0.001) (Table 3). Among mental health care users, there were no significant differences between disability-eligible and age-eligible veterans in the number of VA mental health visits (*p *= 0.45), Medicare mental health visits (*p *= 0.23), and total mental health care visits (*p *= 0.96).

**Table 3:** Adjusted Annual Mental Health Visits between Disability- and Age-Eligible Veteran Patients Using Two-Part Model1

	Disability eligible (reference = age eligible)	
	
	**Any Use**^**2**^	**Level of Use**^**3**^
	
Measure	Odds Ratio (95% CI)	Incidence Rate Ratio (95% CI)
Sample size^4^	15520	Variable

Mental health visits		

VA	1.74 (1.50-2.01)**	0.93 (0.77-1.12)

Medicare	1.46 (1.10-1.95)*	1.27(0.86-1.87)

Total	1.73 (1.52-1.99)**	1.05 (0.85-1.18)

*p < 0.01; **p < 0.001

^1^Adjusted for patient characteristics, zip code level characteristics, year fixed effects, and sampling weights.

^2^This is the 1st part model for two-part models.

^3^This is the second-part model including patients with use > 0.

^4^The sample size for one-part model is 15,520. For second-part model, the sample size varies across measures depending on the number users for the specific services.

## Discussion

This is one of the first studies to compare use of outpatient services in VA and Medicare between disability-eligible and age-eligible veterans among VA primary care users. We found that disability-eligible veterans were more reliant on VA for primary care, specialty care and mental health care than age-eligible veterans following a fixed cohort from FY2001-FY2004. Both groups sought primary care most commonly in VA, which differs from results of a study examining 1997-1999 outpatient care patterns for male, elderly Medicare-eligible veterans from three states in New England [27]. Our finding of greater VA reliance for primary care may have been driven by our selection of a sample of veterans who had one or more primary care visits in VA in FY2000, which may have been influenced by a growing VA emphasis on primary care services [5] in this period.

Dual use of specialty care in VA and Medicare was common (47-50%) and similar in the two groups, while dual use of primary care was somewhat less common and less likely among disability-eligible veterans. We also found that, as expected, disability-eligible veterans had more total (VA+Medicare) primary care and specialty care visits than age-eligible veterans, which was largely driven by more VA visits.

Dual use of specialty care in VA and Medicare may indicate strategic choices about the source of particular types of specialty care by Medicare-eligible veterans, such as having a Medicare cardiologist but a VA pulmonologist. This pattern may be less duplicative than obtaining primary care from two different sources, but still fragments both care and the medical record, which may impact chronic disease management, continuity of care, and quality measurement at provider and system levels. Future research should explore whether disability-eligible and age-eligible veterans differ in the types of specialty care they obtain in VA or Medicare and assess quality outcome effects of fragmentation.

We also found that disability-eligible veterans were more likely to have one or more mental health visits in VA or Medicare than age-eligible veterans, and obtained outpatient mental health care almost exclusively in VA. The greater likelihood of mental health services may reflect a greater burden of mental health impairment among disability-eligible veterans [1,13,18] and these impairments themselves can be the proximal reason for the Medicare disability-eligibility, but there were no differences in the adjusted number of mental health care visits in either system among the subset of disability-eligible and age-eligible veterans who had a visit. Preference for VA mental health services by these veterans may be related to VA's well established mental health programs, or to an emphasis on medication management in Medicare and less time with Medicare mental health providers [48]. This may also reflect the limited mental coverage under Medicare. Veterans returning from recent wars with trauma, post-traumatic stress disorder, or other mental health conditions who were not part of this study given its FY2000 sampling may make similar choices in their preferred source of mental health care in the future, because VA is developing more expertise than community mental health providers in these types of treatment [49-51].

This study has several limitations. This is not a random sample of Medicare eligible veterans in the VA system, though it was determined by trying to balance urban and rural locations. In focusing on Medicare and VA, the study does not include utilization data provided under other insurance coverage, including Medicaid and private insurance, though the emphasis on the disabled population for outpatient care lessens the likely impact of this focus. This study did not include Medicare-eligible veterans who solely relied on Medicare. Further, these results show the experience of Medicare-eligible veterans who used VA primary care in FY2001-FY2004 before the implementation of Medicare Part D, so these results may not generalize to more current experience of veterans facing different choices regarding access to pharmaceuticals. However, VA pharmaceutical prices and policies have remained attractive to veterans thus far. The current medication copayment is $9 for a 30-day supply among veterans who are not eligible for free VA care. The study cohort also precedes the influx of returning veterans from the Afghanistan and Iraq wars, which has resulted in large increases in VA budgets and provision of more specialty care and mental health care aimed at these younger veterans. Further research should assess utilization among these young veterans. Our cohort was somewhat similar to a cohort of Afghanistan and Iraq veterans who used VA in 2001-2005 that were examined in a recent study [52]. Our cohort was older, less likely to be female (3% vs. 13%), more likely to be married (59% vs. 43%), and more likely to be Caucasian (87% vs. 69%) than Afghanistan and Iraq veterans from the prior study. Finally, there could be important unobserved confounding factors that might explain differences between disability- and age-eligible veterans. Despite these limitations, the results of this study are novel, in part because few studies have examined outpatient use of VA and Medicare by disability-eligible veterans. This study provides an important baseline for future research assessing utilizations among returning veterans who use both VA and Medicare systems.

## Conclusions

This study shows greater outpatient care needs among disability-eligible veterans than age-eligible veterans, especially for VA care. This has important implications for care of younger veterans returning from recent wars, who are entering the system in growing numbers [53] and whose health needs are likely to be greater than in prior veteran cohorts [54,55]. VA and Centers for Medicare and Medicaid Services may benefit from establishing care coordination protocols to ensure efficient use of taxpayer resources and smooth transitions for disabled veterans from the Department of Defense and TRICARE through VA care and also accounting for Medicare access under the disability program. These efforts may reduce or avoid some of the historic disparities in care and access experienced by disabled patients, to ensure that these vulnerable patients achieve the best possible access to care and outcomes of care received.

## Competing interests

The authors declare that they have no competing interests.

## Authors' contributions

CFL conceived of the study, obtained grant funding, designed the analyses, interpreted the results, and drafted and revised the manuscript. CLB, JFB, and MLM conceived of the study, obtained grant funding, designed the analyses, interpreted the results, and participated in manuscript preparation. MP cleaned and linked all of the data files, conducted all of the statistical analyses, and participated in manuscript preparation. NS participated in coordination of the study, interpretation of results, and manuscript preparation. All authors read and approved the final manuscript.

## Pre-publication history

The pre-publication history for this paper can be accessed here:

http://www.biomedcentral.com/1472-6963/12/51/prepub
